# Expanding insights into plant rhabdovirus diversity through the discovery of viruses representing 32 putative novel species

**DOI:** 10.1007/s00705-026-06609-1

**Published:** 2026-04-09

**Authors:** Marleen Botermans, P. P. M. de Koning, M. Westenberg, I. P. Adams, K. Ben Mansour, C. Chabi-Jesus, C. de Krom, A. M. Dullemans, R. Festus, A. R. Fowkes, A. Fox, J. Freitas-Astúa, M. Hajizadeh, P. Hellin, D. Knierim, B. Krenz, F. Maclot, I. Malandraki, V. I. Maliogka, P. Margaria, S. Massart, E. T. M. Meekes, W. Menzel, C. Oplaat, C. G. Orfanidou, P. L. Ramos-González, J. W. Roenhorst, V. I.D. Ros, S. E. Seal, G. Silva, G. Silva dos Santos, I. E. Tzanetakis, R. A. A. van der Vlugt, J. van Gemert, C. Varveri, M. Verbeek, S. Winter, A. K. J. Giesbers

**Affiliations:** 1 Netherlands Institute for Vectors, Invasive Plants and Plant Health, National Plant Protection Organization, Netherlands Food and Product Safety Authority (NVWA), Wageningen, The Netherlands; 2 Fera Science Ltd, York, UK; 3 Ecology, Diagnostics and Genetic Resources of Agriculturally Important Viruses, Fungi and Phytoplasmas, Czech Agrifood Research Center, Prague, Czech Republic; 4 Department of Plant Protection, Faculty of Agrobiology, Food and Natural Resources, Czech University of Life Sciences Prague, Prague, Czech Republic; 5 Department of Plant Biology, Swedish University of Agricultural Sciences, Uppsala, Sweden; 6 Applied Molecular Biology Laboratory, Instituto Biológico de São Paulo, Av. Conselheiro Rodrigues Alves, São Paulo, Brazil; 7 Wageningen University and Research, Wageningen, The Netherlands; 8 Natural Resources Institute, University of Greenwich, Central Avenue, Chatham Maritime, Kent, UK; 9 Department of Agricultural Science and Plant Protection, Mississippi State University, Mississippi, USA; 10 EMBRAPA Cassava and Fruits, Cruz das Almas, Bahia, Brazil; 11 Department of Plant Protection, Faculty of Agriculture, University of Kurdistan, Sanandaj, Iran; 12 Plant and Forest Health Unit, Life Sciences Department, Walloon Agricultural Research Center (CRA-W), Gembloux, Belgium; 13 Plant Virus Department, Leibniz Institute DSMZ – German Collection of Microorganisms and Cell Cultures GmbH, Braunschweig, Germany; 14 INRAE & UMR 1332 Biologie du Fruit et Pathologie, University of Bordeaux, Villenave d’Ornon Cedex, France; 15 Laboratory of Virology, Benaki Phytopathological Institute, Kifissia, Greece; 16 Plant Pathology Laboratory, School of Agriculture, Aristotle University of Thessaloniki, Thessaloniki, Greece; 17 Plant Pathology Laboratory, Gembloux Agro-Bio Tech, TERRA, University of Liège, Gembloux, Belgium; 18 Netherlands Inspection Service for Horticulture (Naktuinbouw), Roelofarendsveen, The Netherlands; 19 Department of Entomology and Plant Pathology, University of Arkansas System Division of Agriculture, Fayetteville, 72703 USA

**Keywords:** *Rhabdoviridae*, High-throughput sequencing, Data sharing, Taxonomic diversity, *Betarhabdovirinae*

## Abstract

Plant-infecting rhabdoviruses (family *Rhabdoviridae*, subfamily *Betarhabdovirinae*) include several viruses that cause important crop diseases and are subject to phytosanitary regulation. Despite their agricultural and ecological importance, the diversity of plant rhabdoviruses and their impact on plant health remain poorly understood. Here, we report 32 tentative novel species of plant-infecting rhabdoviruses, identified via high-throughput sequencing and spanning nine established genera. The virus sequences originated from diverse hosts and geographic regions, revealing extensive diversity within the family *Rhabdoviridae*. Several viruses were detected independently in the same host species across multiple countries, demonstrating the practical value of data sharing for confirming host associations and gaining insight into the geographic distribution of these viruses. Our study highlights the underexplored diversity of plant rhabdoviruses and demonstrates the value of coordinated, collaborative virus discovery. With HTS now widely accessible, the challenge has shifted from virus discovery to making sequence data and metadata publicly available, and to conducting the time-consuming biological characterization often deprioritized in favour of viruses with immediate phytosanitary relevance. As a result, many findings remain unreported, leaving valuable data dormant on servers. By sharing genomic data prior to publication, we present an efficient approach to accelerate virus reporting, enable comparative analyses and advance understanding of virus diversity. We hope this collaborative effort will encourage further exploration of plant viruses, including those from hosts without discernable symptoms, supporting virus biology, taxonomy, pest risk assessments, and plant health policies.

**Supplementary Information:**

The online version contains supplementary material available at 10.1007/s00705-026-06609-1.

## Introduction

Rhabdoviruses (family *Rhabdoviridae*) are a diverse group of negative-sense RNA viruses that infect a wide range of organisms, including plants, animals and fungi [1]. Plant-infecting rhabdoviruses are classified within the subfamily *Betarhabdovirinae* and are traditionally recognized morphologically by their characteristic enveloped bacilliform or bullet-shaped particles. Currently, the subfamily *Betarhabdovirinae* comprises 12 genera and 253 species [2], with virus genomes consisting of one- three RNA segments [3, 4]. Transmission generally depends on arthropods, such as aphids, leafhoppers, planthoppers and mites or, in some cases, chytrid fungi, usually with highly specific virus-vector interactions [4, 5].

Rhabdovirus infections may cause vein yellowing, leaf deformation, stunting and other symptoms, whereas asymptomatic infections are also common. Several viruses are associated with diseases and serious economic losses [6] with various viruses being subject to regulatory measures, including cereal chlorotic mottle virus (CCMoV), citrus chlorotic spot virus (CiCSV), eggplant mottled dwarf virus (EMDV), lettuce necrotic yellows virus (LNYV) and potato yellow dwarf virus (PYDV) [7].

Despite the agricultural and ecological relevance of plant rhabdoviruses, their diversity and their impact on plant health remain largely underexplored. In recent years, the number of sequenced plant-associated rhabdoviruses has increased rapidly, largely driven by the use of high-throughput sequencing (HTS) technologies [8–10]. This has led to major taxonomic revisions accepted by the International Committee on Taxonomy of Viruses (ICTV) [2].

In this study, we report 32 tentative new species of plant-infecting rhabdoviruses. These findings, brought together through pre-publication data sharing, aim to enhance our understanding of rhabdovirus diversity, their potential impact on plant health, and their evolutionary relationships within the family *Rhabdoviridae*.

## Materials and methods

This study compiled putative novel plant rhabdovirus sequences through collaborative contributions from 18 institutes and universities. The sequences were derived from 36 samples originating from 4 continents/14 countries representing 28 plant species from 15 botanical families (Table 1). Virus sequences originated from cultivated and wild plants, reference collections as well as a historical herbarium specimen dating back to 1967 (Table 1). For some of the collected samples, virus-like symptoms were observed, yet most appeared asymptomatic. Most samples consisted of leaf tissue and, in some cases, material from plants of the same species was bulked prior to sequencing. All participating institutes/universities performed HTS, but specific protocols differed. A detailed description of each HTS protocol, including the subsequent identification of rhabdovirus sequences, is provided in supplemental File S1. Some participants performed additional investigations using RT-PCR, PCR-Sanger sequencing, bio-assays or transmission electron microscopy. Detailed information per sample is provided in supplemental Table S1.

**Table 1:** Sample information

Plant species	Sample code	Country of origin	Institute^1^	Collection year	Sample type	No. of plants^2^	Tissue
*Achillea millefolium*	6166765	Netherlands	NIVIP	2020	wild plant	20	leaf
*Artemisia vulgaris*	6166992	Netherlands	NIVIP	2020	wild plant	13	leaf
*Capsicum* sp.	36109219 24070172	Netherlands (ex^3^: South Africa) Netherlands (ex: South Africa)	NIVIP NIVIP	2020 2024	crop crop	3 5	fruit fruit
*Clerodendrum thomsoniae*	Prb1	Brazil	IB-SP, Embrapa	2017	ornamental plant	1	leaf
*Dioscorea cayenensis* subsp. *rotundata*	Ogoja	Nigeria	NRI	2019	crop	1	leaf
*Dracaena marginata*	33478182	Netherlands (ex: Costa Rica)	NIVIP	2017	crop	1	leaf
*Fagopyrum esculentum*	FAGO GG-L2	Greece Netherlands	AUTH WUR	2024 2019	crop crop	5 1	leaf leaf
*Ficus microcarpa*	39380696 39720435	Netherlands (ex: China) Netherlands (ex: China)	NIVIP NIVIP	2023 2022	crop crop	1 1	leaf leaf
*Fragaria x ananassa* var. Kurdistan	KM	Iran	UARK	2019	crop	1	leaf
*Geranium* sp.	6166562	Netherlands	NIVIP	2021	wild plant	20	leaf
*Glechoma hederacea*	6166933	Netherlands	NIVIP	2020	wild plant	5	leaf
*Heptapleurum arboricola*	41903396	Netherlands (ex: Costa Rica)	NIVIP	2022	crop	1	leaf
*Heracleum sphondylium*	6165869 6166538	Netherlands Netherlands, Bergerden	NIVIP NIVIP	2021 2021	wild plant wild plant	20 20	leaf leaf
*Laburnum * *x* * watereri*	41310064	Netherlands	NIVIP	2022	crop	1	leaf
*Laburnum x watereri* ‘Vossii’	WAG0454173	Netherlands	NIVIP	1967	historical collection	1	leaf
*Lamium album*	W120	Czech Republic	CARC	2022	wild plant	6	leaf
*Malus* sp.	Z40	Greece	BPI	2018	crop	5	leaf
*Medicago lupulina*	6166415	Netherlands, Bergerden	NIVIP	2021	wild plant	16	leaf
*Mentha* sp.	6166458 40776962	Netherlands Netherlands (ex: Kenya)	NIVIP NIVIP	2021 2022	crop crop	20 1	leaf leaf
*Mentha x gracilis* ‘Ginger Variegata’	32653962	Netherlands	NIVIP	2019	crop	1	leaf
*Pastinaca sativa*	6166546	Netherlands, Bergerden	NIVIP	2021	wild plant	7	leaf
*Pelargonium grandiflorum*	40238259	Netherlands (ex: France)	NIVIP	2023	crop	1	leaf
*Petroselinum crispum*	130948 PV-1489 PV-1508	United Kingdom Germany Germany	FERA DSMZ DSMZ	2021 2024 2025	wild plant crop insect	1 2 1	leaf leaf insect
*Phalaenopsis* ‘White World’	39616419	Netherlands	NIVIP	2019	crop	1	leaf
*Rubus* sp. (bramble)	24/0402	Belgium	CRA-W	2024	wild plant	1	leaf
*Sedum* sp.	42336637	Netherlands (ex: Kenya)	NIVIP	2022	crop	1	leaf
*Stachys palustris*	5909889	Netherlands	NIVIP	2023	wild plant	1	leaf
*Urtica dioica*	WAG084	Netherlands	INRAE-Uliège	2023	wild plant	1	leaf
*Vigna unguiculata*	4H	Nigeria	DSMZ	< 1999	crop	1	leaf

^1^ AUTH, Aristotle University of Thessaloniki; BPI, Benaki Phytopathological Institute; CARC, Czech Agrifood Research Center; CRA-W, Walloon Agricultural Research Centre; DSMZ, German Collection of Microorganisms and Cell Cultures; Embrapa, Empresa Brasileira de Pesquisa Agropecuária; Fera, Fera Science; IB-SP, Instituto Biológico de São Paulo; INRAE, National Research Institute for Agriculture, Food and Environment; NIVIP, Netherlands Institute for Vectors, Invasive Plants and Plant health; NRI, Natural Resources Institute, University of Greenwich; UARK, University of Arkansas; Uliège, University of Liège; WUR, Wageningen University & Research

^2^ When more than one plant was sampled, plants of the same species were pooled prior to RNA-extraction or sequencing

^3^ ex: indicates the country from which the plant material originated (import origin)

### Sequence analyses

Sequences of putative novel rhabdoviruses were imported into Geneious Prime (v 2025.1.2). The open reading frames (ORFs) were predicted using the Geneious Find ORFs functions and translated into amino acid sequences which were subsequently analyzed with NCBI BLASTp (26 November 2025). Lowest e-value with corresponding accession, query coverage and %identity were assessed for each predicted ORF (supplemental Table S1). The intergenic regions were determined following Bejerman [8, 10, 11] (supplemental Table S2).

### Phylogenetic analyses

For rhabdovirus phylogenetic analyses the L protein, encoding the RNA-dependent RNA polymerase (RdRp) is commonly used. Reference sequences of all *Betarhabdovirinae* member species were selected using the ICTV Virus Metadata Resource (VMR_MSL40.v1.20250307) [2] and corresponding L amino acid sequences from NCBI GenBank were imported into Geneious Prime. These reference sequences and those obtained in this study, were aligned using MAFFT (v7.490) [12]. The best-fitting amino acid substitution model (LG + F+I+G4) was determined using ModelFinder [13] as implemented in IQ-TREE 2 (v 2.3.6) [14]. A maximum-likelihood phylogenetic tree was then inferred in IQ-TREE 2 using this model with 10,000 ultrafast bootstrap replicates [15]. The L protein of Puerto Almendras virus (YP_009094394), from the subfamily *Alpharhabdovirinae*, was included as the outgroup. The resulting tree was visualized in TreeViewer (v 2.2.0) [16], transformed into circular style, and clades without putative novel virus species were collapsed for clarity.

### Serratus data mining

To determine whether any of the identified rhabdoviruses were present in the Sequence Read Archive (SRA), their L amino acid sequences were queried using Serratus palmID Viral-RdRp analysis (accessed, August 1, 2025, serratus.io/palmid).

### Transmission electron microscopy

Leaf tissue of *Nicotinana benthamiana* infected with Buckwheat alphacytorhabdovirus (sample GG-L2) was cut in ultra-pure water and a droplet of the sap was applied to a formfar-carbon-coated copper grid (400 mesh; EMS, Hatfield, USA). The grid was stained with 2% Uranyl-acetate and after drying, examined with a JEOL JEM-1400Plus transmission electron microscope.

## Results

The HTS datasets allowed the reconstruction of 39 nearly complete and 2 partial genomic sequences from members of 32 putative rhabdovirus species, none of which showed significant similarity to sequences in GenBank or by data mining with using Serratus. The rhabdovirus sequences were obtained from 36 samples representing 28 plant species across 15 families, collected between 1967 and 2025 from 14 countries (Table 1 and supplemental Table S1). Mixed infections with viruses from the same or other families were detected in 81% (29 out of 36) of the samples (supplemental Table S1).

**Fig. 1:**
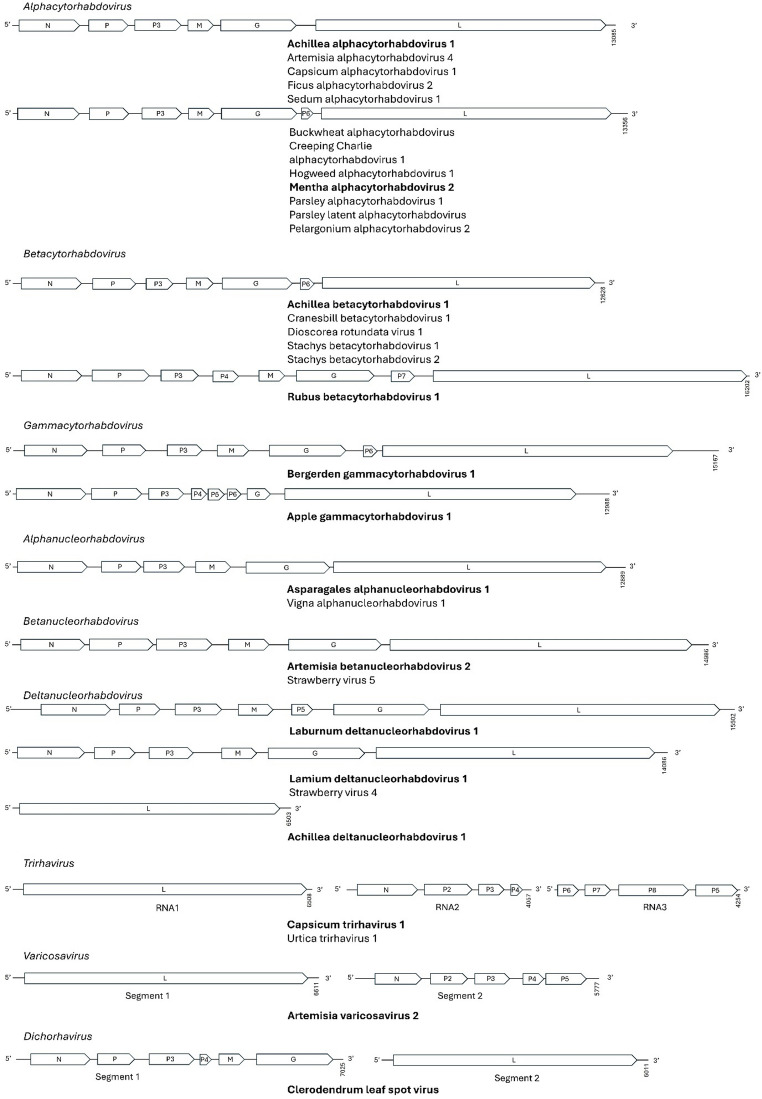
Schematic representation of the genomic organization, shown in reverse polarity, for at least one representative putative species from each genus, indicated in bold. Gene abbreviations: N, nucleoprotein; P, phosphoprotein; P3, putative cell-to-cell movement protein; P4-P8, hypothetical proteins; M, matrix protein; G, glycoprotein; L, RNA-dependent RNA polymerase. All refer to coding sequences (CDS)

**Fig. 2:**
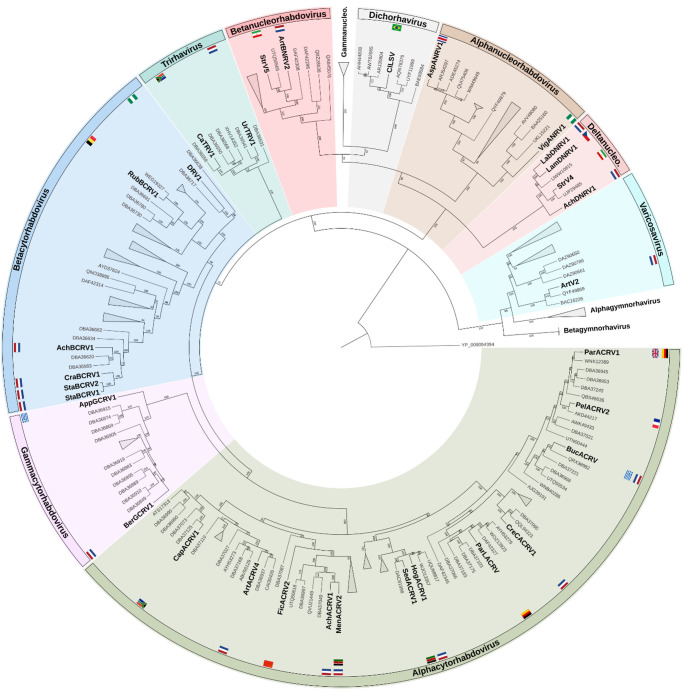
Maximum likelihood phylogenetic tree based on the L (RNA-dependent RNA polymerase; RdRp) amino acid sequences. Bold: acronyms of novel viruses. Not bold: NCBI accession numbers representing reference sequences of known rhabdoviruses. Collapsed: clades without novel virus sequences. The tree was constructed using IQ-TREE 2 using the LG + F+I+G4 substitution model and 10,000 bootstrap replicates. Bootstrap support values indicate the percentage of replicate trees in which the associated clade is recovered, reflecting the robustness of the inferred branching. The L protein of Puerto Almendras virus (YP_009094394) was included as an outgroup. For an expanded version of this tree, with additional details see supplemental Fig S1.

The rhabdovirus genomes were either mono-, bi-, or tri-partite and ranged in size from 6,503 to 16,202 nucleotides (Fig. 1). Based on phylogenetic analyses of the L protein (Fig. 2, supplemental Fig S1), genome organization (Fig. 1), sequence identity to other rhabdovirus sequences and taking into account the ICTV demarcation criteria, the putative novel viruses were tentatively assigned to nine previously established *Betarhabdovirinae* genera: *Alphacytorhabdovirus* (12), *Alphanucleorhabdovirus* (2), *Betacytorhabdovirus* (6), *Betanucleorhabdovirus* (2), *Deltanucleorhabdovirus* (4), *Dichorhavirus* (1), *Gammacytorhabdovirus* (2), *Trirhavirus* (2) and *Varicosavirus* (1) (Fig. 1; Table 2 and supplemental Table S1).

The majority of genome sequences displayed the expected genome organization for their respective genus (Fig. 1). For Achillea deltanucleorhabdovirus 1 only a single contig of 6,503 nucleotides was detect, containing only the *L* gene. In apple gammacytorhabdovirus, the *M* gene is absent and three consecutive ORFs are predicted on its expected position. Furthermore, additional ORFs were present in several viruses interposed between the conserved rhabdovirus structural proteins.All sequences were submitted to GenBank (accession numbers: ON924784, PQ848120, PQ787168-PQ787170, PV555428, PV555429, PV695571, PV933990, PV979719, PX051446-PX051448, PX121434-PX121466, PX550110 and PX550111) and the corresponding raw sequencing reads are available under BioProject PRJNA1344864 in the NCBI Sequence Read Archive (SRA). Detailed information per sample is summarized in supplemental Table S1.

**Table 2:** Tentative novel rhabdoviruses identified in this study and their corresponding hosts

Tentative species name	Virus name	Host(s)	Country of origin
*Alphacytorhabdovirus*			
*Alphacytorhabdovirus achilleae*	Achillea alphacytorhabdovirus 1^1^	*Achillea millefolium*	Netherlands
*Alphacytorhabdovirus betafici*	Ficus alphacytorhabdovirus 2	*Ficus microcarpa* *Ficus microcarpa*	Netherlands (ex: China) Netherlands (ex: China)
*Alphacytorhabdovirus betamenthae*	Mentha alphacytorhabdovirus 2	*Mentha* sp. *Mentha* sp. *Mentha x gracilis* ‘Ginger Variegata’	Netherlands Netherlands (ex: Kenya) Netherlands
*Alphacytorhabdovirus betapelargonii*	Pelargonium alphacytorhabdovirus 2	*Pelargonium grandiflorum*	Netherlands (ex: France)
*Alphacytorhabdovirus betapetroselini*	Parsley latent alphacytorhabdovirus	*Petroselinum crispum*	Germany
*Alphacytorhabdovirus capsici*	Capsicum alphacytorhabdovirus 1	*Capsicum* sp.	Netherlands (ex: South Africa)
*Alphacytorhabdovirus deltaartemisiae*	Artemisia alphacytorhabdovirus 4^2^	*Artemisia vulgaris*	Netherlands
*Alphacytorhabdovirus fagopyrum*	Buckwheat alphacytorhabdovirus	*Fagopyrum esculentum* *Fagopyrum esculentum*	Greece Netherlands
*Alphacytorhabdovirus glechomae*	Creeping Charlie alphacytorhabdovirus 1	*Glechoma hederacea*	Netherlands
*Alphacytorhabdovirus heraclaei*	Hogweed alphacytorhabdovirus 1	*Heracleum sphondylium*	Netherlands
*Alphacytorhabdovirus petroselini*	Parsley alphacytorhabdovirus 1	*Petroselinum crispum*	United Kingdom Germany
*Alphacytorhabdovirus sedii*	Sedum alphacytorhabdovirus 1	*Sedum* sp.	Netherlands (ex: Kenya)
*Betacytorhabdovirus*			
*Betacytorhabdovirus achilleae*	Achillea betacytorhabdovirus 1^1^	*Achillea millefolium*	Netherlands
*Betacytorhabdovirus dioscoreae*	Dioscorea rotundata virus 1	*Dioscorea cayenensis* subsp. *rotundata*	Nigeria
*Betacytorhabdovirus geraniae*	Cranesbill betacytorhabdovirus 1	*Geranium* sp.	Netherlands
*Betacytorhabdovirus stachyos*	Stachys betacytorhabdovirus 1^3^	*Stachys palustris*	Netherlands
*Betacytorhabdovirus betastachyos*	Stachys betacytorhabdovirus 2^3^	*Stachys palustris*	Netherlands
*Betacytorhabdovirus spinirubi*	Rubus betacytorhabdovirus 1	*Rubus* sp. (bramble)	Belgium
*Gammacytorhabdovirus*			
*Gammacytorhabdovirus bergerdensis*	Bergerden gammacytorhabdovirus 1	*Medicago lupulina* *Heracleum sphondylium* *Pastinaca sativa* *Phalaenopsis* ‘White World’	Netherlands Netherlands Netherlands Netherlands
*Gammacytorhabdovirus mali*	Apple gammacytorhabdovirus 1	*Malus* sp.	Greece
*Alphanucleorhabdovirus*			
*Alphanucleorhabdovirus costaricensis*	Asparagales alphanucleorhabdovirus 1	*Dracaena marginata* *Heptapleurum arboricola*	Netherlands (ex: Costa Rica) Netherlands (ex: Costa Rica)
*Alphanucleorhabdovirus vignae*	Vigna alphanucleorhabdovirus 1	*Vigna unguiculata*	Nigeria
*Betanucleorhabdovirus*			
*Betanucleorhabdovirus betaartemisiae*	Artemisia betanucleorhabdovirus 2^2^	*Artemisia vulgaris*	Netherlands
*Betanucleorhabdovirus kurdistanfragariae*	Strawberry virus 5	*Fragaria x ananassa* var. Kurdistan	Iran
*Deltanucleorhabdovirus*			
*Deltanucleorhabdovirus achilleae*	Achillea deltanucleorhabdovirus 1^1^	*Achillea millefolium*	Netherlands
*Deltanucleorhabdovirus kurdistanfragariae*	Strawberry virus 4	*Fragaria x ananassa* var. Kurdistan	Iran
*Deltanucleorhabdovirus laburni*	Laburnum deltanucleorhabdovirus 1	*Laburnum x watereri * *Laburnum x watereri* ‘Vossii’	Netherlands
*Deltanucleorhabdovirus lamii*	Lamium deltanucleorhabdovirus 1	*Lamium album*	Czech Republic
*Trirhavirus*			
*Trirhavirus capsici*	Capsicum trirhavirus 1	*Capsicum* sp.	Netherlands (ex: South Africa)
*Trirhavirus urticae*	Urtica trirhavirus 1	*Urtica dioica*	Netherlands
*Varicosavirus*			
*Varicosavirus betaartemisiae*	Artemisia varicosavirus 2^2^	*Artemisia vulgaris*	Netherlands
*Dichorhavirus*			
*Dichorhavirus piracicabense*	Clerodendrum leaf spot virus	*Clerodendrum thomsoniae*	Brazil

^1^Viruses identified in the same plant sample (*Achillea millefolium*); ^2^Viruses identified in the same (bulked) plant sample (*Artemisa vulgaris*); ^3^Viruses identified in the same (bulked) plant sample (*Stachys palustris*). Abbreviation ex: indicates the country from which the plant material originated (import origin)

**Fig. 3:**
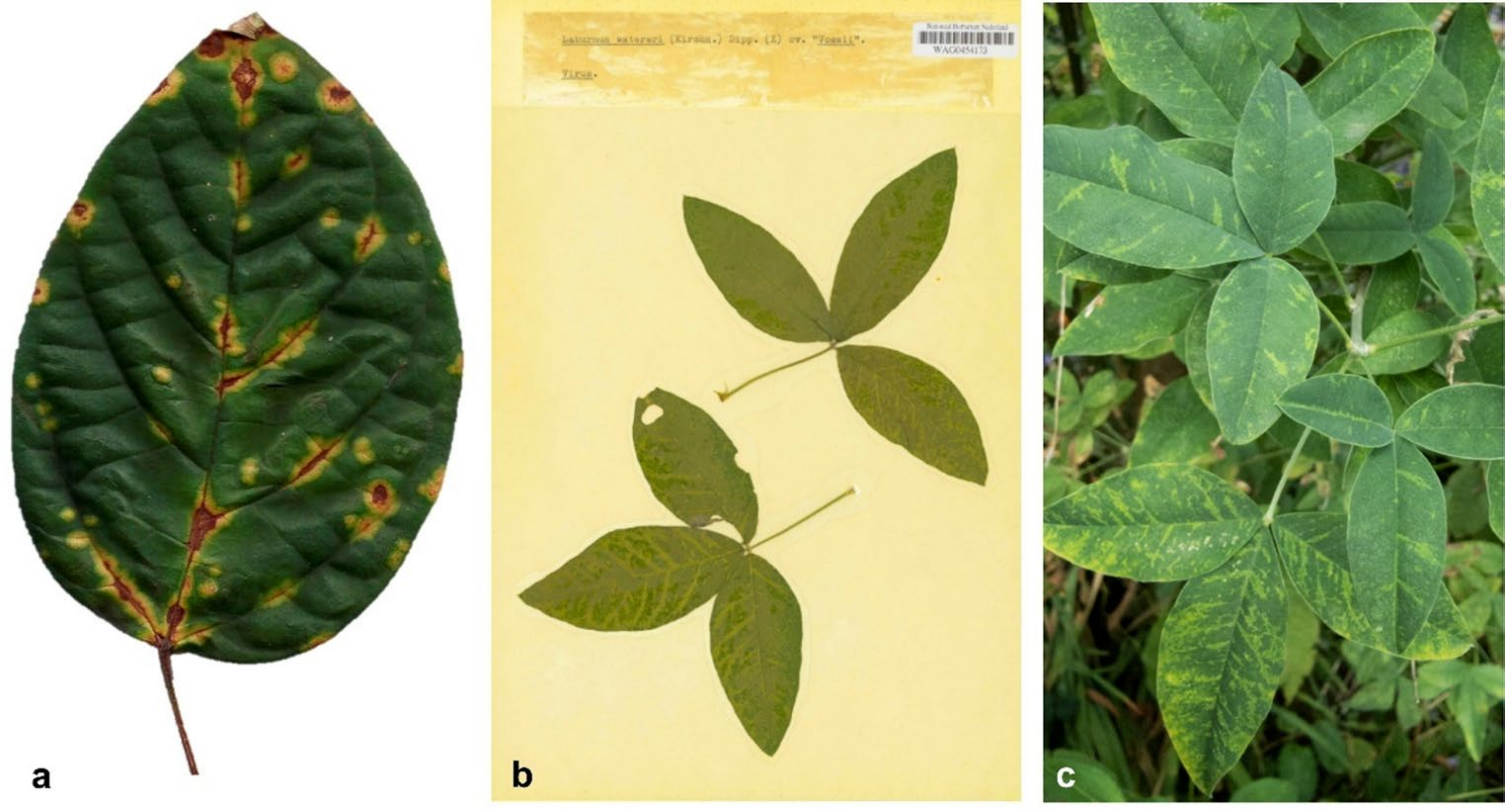
Virus-like symptoms observed in plants infected with putative novel rhabdoviruses. **A**) Chlorotic and necrotic spots on a leaf of a *Clerodendrum thomsoniae* plant (Prb1) infected with Clerodendrum leaf spot virus. **B**) Vein-yellowing and vein-banding in a *Laburnum* x *watereri* herbarium specimen (WAG0454173) from 1967 infected with Laburnum deltanucleorhabdovirus 1. **C**) Vein-yellowing, vein-banding, and mosaic in a living *Laburnum* x *watereri* tree (41310064) infected with Laburnum deltanucleorhabdovirus 1 and Arabis mosaic virus

Virus-like symptoms were observed in 20 out of 36 samples (supplemental Table S1). We have examined which of these samples could potentially be associated with the putative novel viruses. Sixteen of these samples were co-infected with other viruses and were therefore excluded from this examination. Two samples displayed clear virus-like symptoms and were singly infected: *Clerodendrum thomsoniae* (Prb1) showing chlorotic and necrotic spots (Fig. 3a), and *Laburnum* x *watereri* (WAG0454173) displaying vein-yellowing and vein-banding (Fig. 3b). In an additional *Laburnum* x *watereri* sample (41310064), infected with the same putative novel virus, similar symptoms were observed, though it was not in single infection (Fig. 3c). No symptoms were observed in 13 samples, 10 of which were wild plants, while the symptom status was unclear for three samples.

Transmission electron microscopy of leaf tissue from *Nicotiana benthamiana* infected with Buckwheat alphacytorhabdovirus (GG-L2) revealed bacilliform virus particles typical of rhabdoviruses, with approximately 263 nm in length and 93 nm in width (supplemental Fig. S2).

## Discussion

The 32 plant rhabdovirus sequences reported here were independently identified by 18 collaborating institutes/universities, each using different HTS approaches. Most species were identified by only one institute, whereas a few were identified by multiple institutes. The majority of genome sequences displayed the expected rhabdovirus genome organization, although several exceptions were observed, consistent with previously reported variability within the family *Rhabdoviridae* [17]. Our study illustrates not only the diversity of plant rhabdoviruses but also the practical benefits of pre-publication data sharing for accelerating virus discovery, characterization, and contextualization. This collaborative approach reduced duplication of efforts, offered early insights into host range, geographical distribution and potential symptom associations, all of which support taxonomy and pest risk assessments [18–21]. Such coordinated efforts also increase transparency and encourage data reuse, thereby advancing the field of plant virology.

Since most samples with virus-like symptoms were coinfected with other viruses, it was not possible to determine whether the identified rhabdoviruses are associated with symptoms. As Fox [22] emphasizes, establishing a causal relationship in plant virology is often challenging, particularly in mixed infections. Moreover, in several cases, it remains uncertain whether the virus-like symptoms were induced by viruses at all or by other factors. Further biological characterization studies, ideally using singly infected plants in controlled conditions, will therefore be required to determine potential etiological relationships.

Nevertheless, two examples suggest potential virus-disease associations involving singly-infected samples. Laburnum deltanucleorhabdovirus 1 was detected in two *Laburnum* × *watereri* samples. The viral sequence was found both in a symptomatic herbarium specimen collected in 1967 where it occurred as a single infection, and in a living *Laburnum* × *watereri* tree co-infected with Arabis mosaic virus (*Nepovirus arabis*). Both plants exhibited similar virus symptoms of vein-yellowing and vein-banding with the living tree also showing mosaic patterns (Fig. 3a, b). Historical records by Masters [23] in 1877, van Katwijk [24] in 1953, and transmission electron microscopy observations of rhabdovirus-like particles by Cooper [25] support a long-observed potential link between vein-banding and mosaic symptoms in *Laburnum* and virus infection. This case also demonstrates the value of integrating historical herbarium material with modern molecular techniques. Similarly, Clerodendrum leaf spot virus was detected in singly infected *Clerodendrum thomsoniae* plants (data not shown), exhibiting chlorotic leaf spots (Fig. 3c), indicating potential pathogenicity of this virus. For both examples additional studies are needed to establish potential etiological relationships, ideally following the integrated approaches of Fontdevila Pareta et al. and Fox et al. [20, 22], including but not limited to screening of both asymptomatic and symptomatic plants in ecosystems and inoculation in controlled conditions.

In addition to 20 symptomatic plant samples, our study included 13 asymptomatic samples in which putative novel rhabdoviruses were identified. Many of these asymptomatic samples originated from virus reservoir surveys in wild plants, suggesting that numerous rhabdoviruses may not induce obvious symptoms in their hosts [10]. This is consistent with reports from other virus families, where asymptomatic infections are also frequently observed [26–28]. Together, these findings illustrate the high viral diversity that can infect apparently healthy plants within and outside agricultural ecosystems and supports the view that large-scale virus reservoir studies are important for biosecurity as they provide insights into the host range of viruses and allow better identification and allocation of the species potentially posing a phytosanitary risk [19].

### Same rhabdovirus repeatedly detected in the same host species

Pre-publication data sharing enabled the early detection and cross-validation of potential virus–host associations and revealed that certain putative virus species are found across different countries. For example, parsley alphacytorhabdovirus 1 was independently detected in *Petroselinum crispum* (parsley) samples from the United Kingdom and Germany. Similarly, buckwheat alphacytorhabdovirus was identified in *Fagopyrum esculentum* (buckwheat) growing in habitat-enhanced field margins in Greece and the Netherlands. In addition, strawberry virus 4 and strawberry virus 5 were detected in the USA and Iran, suggesting a broad geographic presence. Ficus alphacytorhabdovirus 2 was detected in two *Ficus microcarpa* plants imported separately from China, cross-validating its host and distribution. Furthermore, Mentha alphacytorhabdovirus 2 was detected in three samples, namely from two cultivated and one wild *Mentha* species from both the Netherlands and Kenya. These examples highlight the practical value of data sharing, which allowed the independent identification of similar virus genomes in the same host across multiple countries, suggesting these viruses have been circulating for a long time or spreading between countries, for example through international trade.

### Multiple rhabdoviruses infecting the same host species

In some plant samples, multiple distinct rhabdoviruses co-occurred. Stachys betacytorhabdovirus 1 and Stachys betacytorhabdovirus 2 were found in a single *Stachys palustris* plant (sample 5909889), while four distinct alphacytorhabdoviruses were identified in bulked *Artemisia vulgaris* (sample 6166992): Artemisia alphacytorhabdovirus 1–4. Similarly, in bulked sample *Achillea millefolium* (sample 6166765), both Achillea alphacytorhabdovirus 1 and Achillea betacytorhabdovirus 1 were identified, as well as Achillea deltanucleorhabdovirus 1, although only its *L* gene was assembled. These observations highlight the substantial rhabdovirus diversity that can exist within a single host.

### Same rhabdovirus in different host species

Two rhabdoviruses were identified in more than one host species. Asparagales alphanucleorhabdovirus 1 was identified in a *Heptapleurum arboricola* and a *Dracaena marginata* plant, both imported from Costa Rica. Although both plant species belong to the same order (Asparagales), they are members of different families. Similarly, Bergerden gammacytorhabdovirus was identified in three asymptomatic wild species from a single location (Bergerden) and in a symptomatic, cultivated *Phalaenopsis* orchid. These findings suggest that both viruses may be transmitted by a polyphagous vector and that further screening may reveal additional host plant species, as observed for Physostegia chlorotic mottle virus (PhCMoV; *Alphanucleorhabdovirus physostegiae*) [18, 29].

### Hidden diversity of plant rhabdoviruses

In the past decade, many plant rhabdoviruses have been identified through diagnostic testing, virus reservoir studies and mining of plant transcriptome database studies [8, 10]. However, as with other virus families, many findings are not being formally reported due to time constraints and because priority is often given to viruses or virus groups with clear phytosanitary impact [19, 20]. Our data-sharing-based approach led to the collective identification and publication of 32 putative novel species, underscoring the hidden diversity of this virus group.

Bejerman, et al. [8] reported 27 novel rhabdoviruses through SRA mining, roughly half of which were (putative) cytorhabdoviruses. Similarly, 63% (20 out of 32) of the putative novel rhabdoviruses presented in our study, not identified from the SRA but from actual plant samples, were also cytorhabdoviruses (including alpha-, beta- and gammacytorhabdoviruses). This suggests a rich, but underexplored diversity within this cytorhabdoviruses. However, it is important to note that a large diversity may also exist in other rhabdovirus groups but that this diversity is yet uncovered for example due to under sampling. Gymnosperm-infecting alpha- and betagymnorhavirus, for instance, are likely underrepresented, as gymnosperms tend to be sampled less than herbaceous plant species [8].

This study accounts for nearly 12.6% of the currently known plant rhabdoviruses species and makes a substantial contribution to the family diversity.

### Virus discovery versus biological characterization in the HTS-era

With HTS now available to many labs, the challenge has shifted from virus discovery to the biological characterisation of these putative new viruses. This is due to the associated time-consuming efforts of biological characterisation, with priority typically given to findings with clear crop/plant health or phytosanitary impacts, leaving other findings unreported and dormant on servers [19, 30]. In addition, large amounts of neglected or unused data await secondary analysis and repurposing. Bejerman, et al. [31] predicted that the increasing use of HTS would result in the identification of many more novel viruses with negative-sense and ambisense RNA, including members of the family *Rhabdoviridae*, which is underlined by the 32 novel viruses described here. Although only limited biological, epidemiological and contextual data were available for most of the putative novel viruses in our study, we believe that reporting our findings will encourage other researchers to examine their dormant sequences and datasets. Additionally we hope it will inspire virus reservoir studies, including on asymptomatic plants, and prompt researchers to make their findings publicly available. This would increase our knowledge on host range, distribution, vectors, symptomatology, phytosanitary risks and general understanding of virus epidemiology.

Beyond motivating individual research efforts, our study shows the value of pre-publication data sharing as an important part of plant-health preparedness. Such sharing supports regional and global cooperation and rapid response and is similar to frameworks like ‘disaster plant pathology’ [32], the global crop disease surveillance system proposed by Carvajal-Yepes et al. [33], and parallel initiatives in animal and human virology, such as the Global Virus Network (https://gvn.org/).

In this sense, our work goes beyond filling taxonomic gaps and may contribute to informing the development of more coordinated and responsive approaches for plant-virus monitoring in the future.

## Conclusions

Our study highlights the underexplored diversity of plant rhabdoviruses and demonstrates the value of coordinated, collaborative virus discovery. Through pre-publication data sharing, we offer an efficient approach to accelerate the reporting of tentative novel viruses and deepen our understanding of virus diversity. Even when contextual information is limited, making such data publicly available can provide broader insights into plant virus diversity. It also facilitates comparisons across findings, supports the development of diagnostic tools, and informs plant health policy. We hope this study will encourage further exploration and reporting of plant viruses.

## Supplementary Information

Below is the link to the electronic supplementary material.

Supplemental File S1 (DOCX 44.0 KB)

Supplemental Figure S1. Maximum likelihood phylogenetic tree based on the L (RNA-dependent RNA polymerase; RdRp) amino acid sequences. Bold: names of novel viruses. Not bold: NCBI accession numbers representing reference sequences of known rhabdoviruses. Collapsed: clades without novel virus sequences. Colored boxes: identical colors correspond to the same species. Flags mark the country of origin. The tree was constructed using IQ-TREE 2 using the LG+F+I+G4 substitution model and 10,000 bootstrap replicates. Bootstrap support values indicate the percentage of replicate trees in which the associated clade is recovered, reflecting the robustness of the inferred branching. The L protein of Puerto Almendras virus (YP_009094394) was included as an outgroup. (PNG 1.79 MB)

Supplemental Figure S2. Bacilliform-shaped particle characteristic of rhabdoviruses observed by transmission electron microscopy in Nicotiana benthamiana infected with Buckwheat alphacytorhabdovirus (sample GG-L2).

Supplemental Table S1 (XLSX 32.0 KB)

Supplemental Table S2. Identified 3' end mRNA, Intergenic spacer, 5' end mRNA from each viral sequences. Empty cells: indicate that either no corresponding sequence was identified. Hatched cells: the intergenic spacer and 5′-end mRNA at the 3′ terminus of the genomic segment, which were not examined (i.e., downstream of the last ORF). Asterisk (*): truncated G gene.
