# Supplementary material for: Expanding insights into plant rhabdovirus diversity through the discovery of viruses representing 32 putative novel species
Source: Arch Virol. 2026 Apr 9;171(5):156. doi: 10.1007/s00705-026-06609-1 (PMC13061796; doi:10.1007/s00705-026-06609-1)
Supplement: Supplementary file 1 — Supplemental File S1 (DOCX 44.0 KB) [file 705_2026_6609_MOESM1_ESM.docx]

# Supplemental file S1 HTS protocols

### NIVIP (NL)

Samples were analyzed with HTS as previously described [1]. Total RNA was extracted from approximately 1g of leaf tissue using the RNeasy Plant Mini Kit (Qiagen, Venlo, the Netherlands). Ribosomal RNA was depleted with the Ribo-Zero rRNA Removal Kit (Illumina, Eindhoven, the Netherlands) or the QIAseq FastSelect – rRNA Plant Kit (Qiagen), followed by library preparation with the NEBNext Ultra or Ultra II Directional RNA library Prep Kit (New England Biolabs, Ipswich, MA, USA). Libraries were sequenced on a NextSeq500 or NovaSeq 6000 (Illumina), generating 150 nt paired-end reads.

The resulting reads were trimmed and *de novo* assembled using CLC Genomic workbench (Qiagen) standard tools. Consensus sequences (>100 nt; read depth >10) from the *de novo* assemblies, were analyzed using MegaBLAST and DIAMOND [2] with locally installed NCBI nr/nt and nr databases respectively. Visualizations of BLAST results were carried out in Krona with a bitscore threshold of 25 [3]. Putative viral contigs were analysed in Geneious Prime (v 2025.1.2).

### AUTH (GR)

Total nucleic acid was extracted from approximately 0.5 g of leaf tissue using the Plant/Fungi Total RNA Purification Kit (Norgen Biotek, Thorold, ON, Canada) according to the manufacturer’s protocol. DNase-treatment was carried out using the RNase-Free DNase I Kit (Norgen Biotek). Library preparation was carried out with the TruSeq Stranded Total RNA with Ribo-Zero Plant Kit (Illumina, city, country) as per the manufacturer’s protocol. Sequencing was conducted on a NovaSeq X (Illumina) generating 100 nt paired-end reads, at Macrogen (Seoul, South Korea). The resulting reads were quality assessed using FastQC [4] and trimmed using PRINSEQ++ [5], and host sequences were removed by mapping the reads to a *F. esculentum* genome (<https://buckwheat.kazusa.or.jp/index.html>) using Bowtie2 [6]. Remaining reads were *de novo* assembled using Spades [7] with default parameters. The resulting contigs were analyzed using BLASTn/BLASTx against the NCBI nr/nt and nr databases.

### BPI (GR)

Total RNA was extracted from 80-100 mg of liquid nitrogen homogenized leaf tissue with a CTAB protocol [8] followed by treatment with Recombinant DNase I (Takara Bio, Kusatsu, Shiga, Japan). Ribosomal RNA was depleted using the Ribo-Zero rRNA Removal Kit (Illumina, San Diego, USA. Library preparation using TruSeq RNA Library Prep Kit v2 (Illumina, San Diego, USA) (omitting poly-A selection step) and sequencing on a NextSeq550 (Illumina) platform were performed at the Biomedical Research Foundation Academy of Athens (BRFAA, Greece), generating 75 nt single-end reads. Reads were trimmed, duplicate reads were removed and *de novo* assembly was performed with Geneious Prime (v 2019) software. Contigs that mapped to the host genome (GenBank accession number GCA_002114115.1) were subtracted and remaining contigs were analyzed using BLASTn or BLASTx. Putative viral contigs were further analyzed with Geneious Prime.

### CARC (CZ)

Total RNA was extracted from approximately 600 mg of leaf tissue (100 mg per plant from six individual plants: *Artemisia vulgaris, Conium maculatum, Lamium album, Malva sylvestris, Rumex crispus, and Urtica dioica*), using the RNeasy Plant Mini kit (Qiagen, Hilden, Germany), following the manufacturer’s instructions. A sequencing library was prepared on an RNA pool using the Illumina DNA Prep Kit (Illumina, USA) including ribosomal RNA depletion with the QIAseq FastSelect – rRNA Plant Kit (Qiagen, Hilden, Germany). First-strand cDNA synthesis was performed using the Maxima H Minus Reverse Transcriptase (Thermo Fisher Scientific, USA), and double-stranded cDNA was generated with the NEBNext Ultra II Non-Directional RNA Second Strand Synthesis Module (New England Biolabs, Frankfurt, Germany). The library was sequenced on a NextSeq 2000 (Illumina) at the Leibniz Institute DSMZ (Braunschweig, Germany), generating 150 nt paired-end reads. Raw reads were quality-trimmed using BBduk, normalized using BBNorm, and host sequences were removed by mapping the reads to sequences from the corresponding plant species. *De novo* assembly was performed in Geneious Prime (v 2025.1.2) and the resulting first 1000 contigs were subjected to BLASTn/BlastTpagainst a custom virus and viroid reference database downloaded from NCBI. Putative viral contigs were further analyzed and curated in Geneious Prime.

### CRA-W (BE)

Approximately 100 mg of symptomatic leaf tissue was ground in GH+ buffer and extracted using the RNeasy Plant Mini Kit (Qiagen, Antwerp, Belgium) as described in [9]. Ribosomal RNA was depleted with the Ribo-Zero rRNA Removal Kit (Illumina, Cambridge, UK) followed by library preparation with the TruSeq Stranded Total RNA kit (Illumina, Cambridge, UK). The library was sequenced on a NovaSeq 6000 (Illumina, Cambridge, UK), generating 150 nt paired-end reads. Library preparation and sequencing were performed at GIGA (University of Liege, Belgium). Virus detection was performed using a bioinformatic pipeline build on the EU Galaxy server (usegalaxy.eu). Briefly, reads were trimmed using BBduk [10] and assembled using rnaviralSPAdes [11]. Resulting contigs were then searched for viral sequences using BLASTn and tBLASTx against GenBank viral RefSeq sequences (accessed June 2024). Putative viral contigs were then manually compared to NCBI nr/nt database using default BLASTn option. Mapping of the reads was performed using BWA-MEM2 [12], removing duplicated sequences using MarkDuplicates [13], and analyzing reads depths with Mosdepth [14].

### DSMZ (DE)

Total RNA was extracted from approximately 0.1 g of plant material using the Spectrum Plant Total RNA kit (Sigma-Aldrich, St. Louis, Missouri, USA) or the RNeasy Plant Mini Kit (Qiagen, Hilden, Germany), or from 10 individuals of *Dysaphis crataegi* using a CTAB-based method. Libraries were prepared with the NEBNext Ultra II RNA Library Prep Kit for Illumina (New England Biolabs, Ipswich, MA, USA), including a ribosomal RNA depletion using the QIAseq FastSelect -rRNA Plant Kit (Qiagen). Libraries were sequenced on a NextSeq2000 (Illumina) generating 150 nt paired-end reads. The resulting reads were analyzed in Geneious Prime (v 2025.1.2, Dotmatics), using a custom bioinformatic pipeline as previously described [15].

### IB, EMBRAPA (BR)

Total RNA was isolated from approximately 0.5 g of symptomatic Clerodendrum leaves. Leaf pieces were ground in the presence of liquid nitrogen, and total RNA extracts were obtained using TRIZOL® Reagent according to the manufacturer’s recommendation (Life Technologies, Foster City, CA, USA). RNA quantification and A260/A280 ratios were estimated using the NanoDrop ND-8000 micro-spectrophotometer (Thermo Scientific, Waltham, MA, USA). Integrity of samples was verified by a Bioanalyser 2100 device (Agilent Technologies, Santa Clara, CA, USA). The RNA extract was sent to the Animal Biotech Laboratory at Escola Superior de Agricultura Luiz de Queiroz, University of São Paulo (Piracicaba, SP, Brazil) for high-throughput sequencing. Library preparation was carried out using the TruSeq Stranded Total RNA (Ribo-Zero Gold) kit (Illumina, San Diego, CA, USA). The Liberary was sequenced on a HiSeq 2500 (Illumina) generating 125 nt paired-end reads. Adapter sequences and low-quality bases were removed from the reads using Trimmonatic [16], followed by a *de novo* assembly using SPAdes [17] and Trinity [18], all implemented in the Galaxy platform v.25.03 [19]. Contigs producing the best BLASTx hits (E-value score ~ 0) with the RNA1 and RNA2 molecules of known dichorhaviruses were selected for further detailed analyses.

### FERA (UK)

The sample was analysed as previously described in Fowkes, et al. [20]. Total RNA was extracted from approximately 0.1 g of leaf tissue using the RNeasy Mini Kit (Qiagen, Manchester, UK), including the optional on column DNase treatment , following the manufacturer's instructions. The RNA was then ribosome depleted using the Ribo-Zero Plant Leaf Kit (Illumina, Cambridge, UK), followed by library preparation with the TruSeq Stranded RNA library Prep Kit (Illumina, Cambridge, UK), as per manufacturer’s instructions. The library was sequenced on a MiSeq (Illumina) using a MiSeq Reagent Kit v3 (600 cycle) (Illumina, Cambridge, UK), generating 300 nt paired-end reads.

The obtained reads were analysed using the ‘Angua’ pipeline described in Fowkes, et al. [20]. Sequences were trimmed to remove low-quality nucleotides from the 3′ end, using a Phred score threshold of 20 using Sickle [21], and *de novo* assembled using Trinity [22]. Contigs ≥ 200 nt were subject to a BLASTn+ search against the complete NCBI GenBank nr/nt database, and contigs with a length ≥ 1000 nt were subject to a BLASTx+ search against the complete NCBI GenBank nr database [23]. Viral contigs were then extracted using MEGAN community edition [24].

### INRAE (FR) – ULiège (BE)

dsRNA was extracted from 750 mg of leaf tissue, as described in Marais, et al. [25]. Library preparation was performed at GIGA (University of Liege, Belgium) with the NEBNext Ultra II DNA Library Prep Kit (New England Biolabs, Ipswich, MA, USA). Libraries were sequenced on a NovaSeq 6000 (Illumina), generating 150 nt paired-end reads. The resulting reads were trimmed using BBDuk ([10] (Quality score 25, minimum 30 nt), merged, duplicated reads removed, and finally *de novo* assembled using rnaviralSPAdes on the Galaxy server (usegalaxy.org). Contigs were analysed using BLASTn and BLASTx with the NCBI nr/nt and nr databases. Viral BLASTx hits (conservative e-value of e-20 cutoff) were isolated for further analyses using Geneious Prime (v 2024.0.3). Viral genomes were obtained by assembling the contigs and then mapping the reads to these contigs (Geneious mapper, 3 iterations, medium-low sensitivity, 5% mismatches and 5% gaps allowed).

### NRI (UK)

Total RNA was extracted from 0.1 g of leaf tissue using a modified CTAB method combined with the RNeasy Plant Mini Kit (Qiagen, Hilden, Germany) [26]. After DNAse treatment of total RNA, Ribosomal RNA was removed using a RiboMinus Plant Kit for RNA-Seq (ThermoFisher Scientific, Waltham, MA, USA). The library was then prepared using a simplified protocol (no enrichment in poly-A RNA) with the TruSeq Stranded mRNA kit (Illumina, San Diego, CA, USA). The library was sequenced on a NextSeq500 (Illumina), generating 75 nt paired-end reads. After primary quality check, the obtained reads were paired, merged and the duplicates eliminated using Geneious software version 11.0.4. (Biomatters, Auckland, New Zealand). Reads were further assembled in contigs by SPAdes software embedded in Geneious [17]. Annotation of contigs was performed using either BLASTn or BLASTx algorithm based on NCBI nt/nr and Viral Proteins databases. The contigs with a viral hit were further analysed by mapping the reads on the contigs using the Geneious mapper.

### UARK (US)

The sample preparation and HTS was performed as previously described [27] and sequenced on a NextSeq 500 or 550 (Illumina) at the Center for Genomics and Proteomics (Oklahoma State University, Stillwater, OK), generating 75 nt pair-end reads. Analysis of the reads was performed using VirFind [28]. Reads were reference assembled to the viral contigs, assigned by VirFind, in CLC Genomics Workbench (v24.0 (Qiagen)). Consensus sequences of these mappings were analyzed using BLASTn and BLASTx with the NCBI GenBank nr/n and nr databases.

### WUR (NL)

Samples were analyzed with HTS as previously described in [29]. Total RNA was extracted from approximately 0.1 mg of leaf tissue using the RNeasy Plant Mini Kit (Qiagen, Venlo, The Netherlands). HTS sequencing was performed by BaseClear (Leiden, The Netherlands) and included ribosomal RNA depletion using the Ribo-Zero rRNA Removal Kit (Illumina, Eindhoven, The Netherlands), followed by library preparation with the TruS Stranded Total RNA Kit (Illumina, Eindhoven, The Netherlands). Libraries were sequenced on a NovaSeq 6000 (Illumina), generating 150 nt paired-end reads. The resulting reads were trimmed and *de novo* assembled using CLC Genomics Workbench (v11.0.2 (Qiagen)) standard tools. Consensus sequences (>400 nt; read depth >100) were analyzed using BLASTn and BLASTx against the NCBI GenBank nr/n and nr databases.

References

1. EPPO (2024) Addendum – New supporting information for PM 7/151 Considerations for the use of high throughput sequencing in plant health diagnostics. EPPO Bulletin 54:253

2. Buchfink B, Xie C, Huson DH (2015) Fast and sensitive protein alignment using DIAMOND. Nature methods 12:59-60

3. Ondov BD, Bergman NH, Phillippy AM (2011) Interactive metagenomic visualization in a Web browser. BMC bioinformatics 12:1-10

4. Andrews S (2010) FastQC: a quality control tool for high throughput sequence data. Babraham Bioinformatics. 2010.

5. Cantu VA, Sadural J, Edwards R (2019) PRINSEQ++, a multi-threaded tool for fast and efficient quality control and preprocessing of sequencing datasets.

6. Langmead B, Salzberg SL (2012) Fast gapped-read alignment with Bowtie 2. Nature methods 9:357-359

7. Prjibelski A, Antipov D, Meleshko D, Lapidus A, Korobeynikov A (2020) Using SPAdes de novo assembler. Current protocols in bioinformatics 70:e102

8. Gambino G, Perrone I, Gribaudo I (2008) A rapid and effective method for RNA extraction from different tissues of grapevine and other woody plants. Phytochemical Analysis 19:520-525

9. Botermans M, Van de Vossenberg B, Verhoeven JTJ, Roenhorst J, Hooftman M, Dekter R, Meekes E (2013) Development and validation of a real-time RT-PCR assay for generic detection of pospiviroids. Journal of virological methods 187:43-50

10. Bushnell B, Rood J, Singer E (2017) BBMerge – Accurate paired shotgun read merging via overlap. PLOS ONE 12:e0185056

11. Meleshko D, Hajirasouliha I, Korobeynikov A (2021) coronaSPAdes: from biosynthetic gene clusters to RNA viral assemblies. Bioinformatics 38:1-8

12. Vasimuddin M, Misra S, Li H, Aluru S (2019) Efficient Architecture-Aware Acceleration of BWA-MEM for Multicore Systems. In: 2019 IEEE International Parallel and Distributed Processing Symposium (IPDPS), pp 314-324

13. McKenna A, Hanna M, Banks E, Sivachenko A, Cibulskis K, Kernytsky A, Garimella K, Altshuler D, Gabriel S, Daly M, DePristo MA (2010) The Genome Analysis Toolkit: a MapReduce framework for analyzing next-generation DNA sequencing data. Genome Res 20:1297-1303

14. Pedersen BS, Quinlan AR (2017) Mosdepth: quick coverage calculation for genomes and exomes. Bioinformatics 34:867-868

15. Zindović J, Čizmović M, Vučurović A, Margaria P, Škorić D (2024) Increased Diversity of Citrus Tristeza Virus in Europe. Plant Disease 108:1344-1352

16. Bolger AM, Lohse M, Usadel B (2014) Trimmomatic: a flexible trimmer for Illumina sequence data. Bioinformatics 30:2114-2120

17. Bankevich A, Nurk S, Antipov D, Gurevich AA, Dvorkin M, Kulikov AS, Lesin VM, Nikolenko SI, Pham S, Prjibelski AD, Pyshkin AV, Sirotkin AV, Vyahhi N, Tesler G, Alekseyev MA, Pevzner PA (2012) SPAdes: a new genome assembly algorithm and its applications to single-cell sequencing. J Comput Biol 19:455-477

18. Haas BJ, Papanicolaou A, Yassour M, Grabherr M, Blood PD, Bowden J, Couger MB, Eccles D, Li B, Lieber M, MacManes MD, Ott M, Orvis J, Pochet N, Strozzi F, Weeks N, Westerman R, William T, Dewey CN, Henschel R, LeDuc RD, Friedman N, Regev A (2013) De novo transcript sequence reconstruction from RNA-seq using the Trinity platform for reference generation and analysis. Nature Protocols 8:1494-1512

19. Community TG (2024) The Galaxy platform for accessible, reproducible, and collaborative data analyses: 2024 update. Nucleic Acids Research 52:W83-W94

20. Fowkes AR, McGreig S, Pufal H, Duffy S, Howard B, Adams IP, Macarthur R, Weekes R, Fox A (2021) Integrating high throughput sequencing into survey design reveals turnip yellows virus and soybean dwarf virus in pea (Pisum sativum) in the United Kingdom. Viruses 13:2530

21. Joshi N, Fass J (2011) Sickle: A sliding-window, adaptive, quality-based trimming tool for FastQ files (Version 1.33) [Software].

22. Grabherr MG, Haas BJ, Yassour M, Levin JZ, Thompson DA, Amit I, Adiconis X, Fan L, Raychowdhury R, Zeng Q (2011) Full-length transcriptome assembly from RNA-Seq data without a reference genome. Nature biotechnology 29:644-652

23. Camacho C, Coulouris G, Avagyan V, Ma N, Papadopoulos J, Bealer K, Madden TL (2009) BLAST+: architecture and applications. BMC bioinformatics 10:421

24. Huson DH, Beier S, Flade I, Górska A, El-Hadidi M, Mitra S, Ruscheweyh H-J, Tappu R (2016) MEGAN community edition-interactive exploration and analysis of large-scale microbiome sequencing data. PLoS computational biology 12:e1004957

25. Marais A, Faure C, Bergey B, Candresse T (2018) Viral double-stranded RNAs (dsRNAs) from plants: alternative nucleic acid substrates for high-throughput sequencing. Viral metagenomics: methods and protocols:45-53

26. Bömer M, Rathnayake AI, Visendi P, Sewe SO, Sicat JPA, Silva G, Kumar PL, Seal SE (2019) Tissue culture and next-generation sequencing: A combined approach for detecting yam (Dioscorea spp.) viruses. Physiol Mol Plant Pathol 105:54-66

27. Villamor D, Keller K, Martin R, Tzanetakis I (2022) Comparison of high throughput sequencing to standard protocols for virus detection in berry crops. Plant disease 106:518-525

28. Ho T, Tzanetakis IE (2014) Development of a virus detection and discovery pipeline using next generation sequencing. Virology 471:54-60

29. Hammond J, Adams IP, Fowkes AR, McGreig S, Botermans M, van Oorspronk JJ, Westenberg M, Verbeek M, Dullemans AM, Stijger CC (2021) Sequence analysis of 43‐year old samples of Plantago lanceolata show that Plantain virus X is synonymous with Actinidia virus X and is widely distributed. Plant Pathology 70:249-258
