# Supplementary figures and images for: Expanding insights into plant rhabdovirus diversity through the discovery of viruses representing 32 putative novel species
Source: Arch Virol. 2026 Apr 9;171(5):156. doi: 10.1007/s00705-026-06609-1 (PMC13061796; doi:10.1007/s00705-026-06609-1)

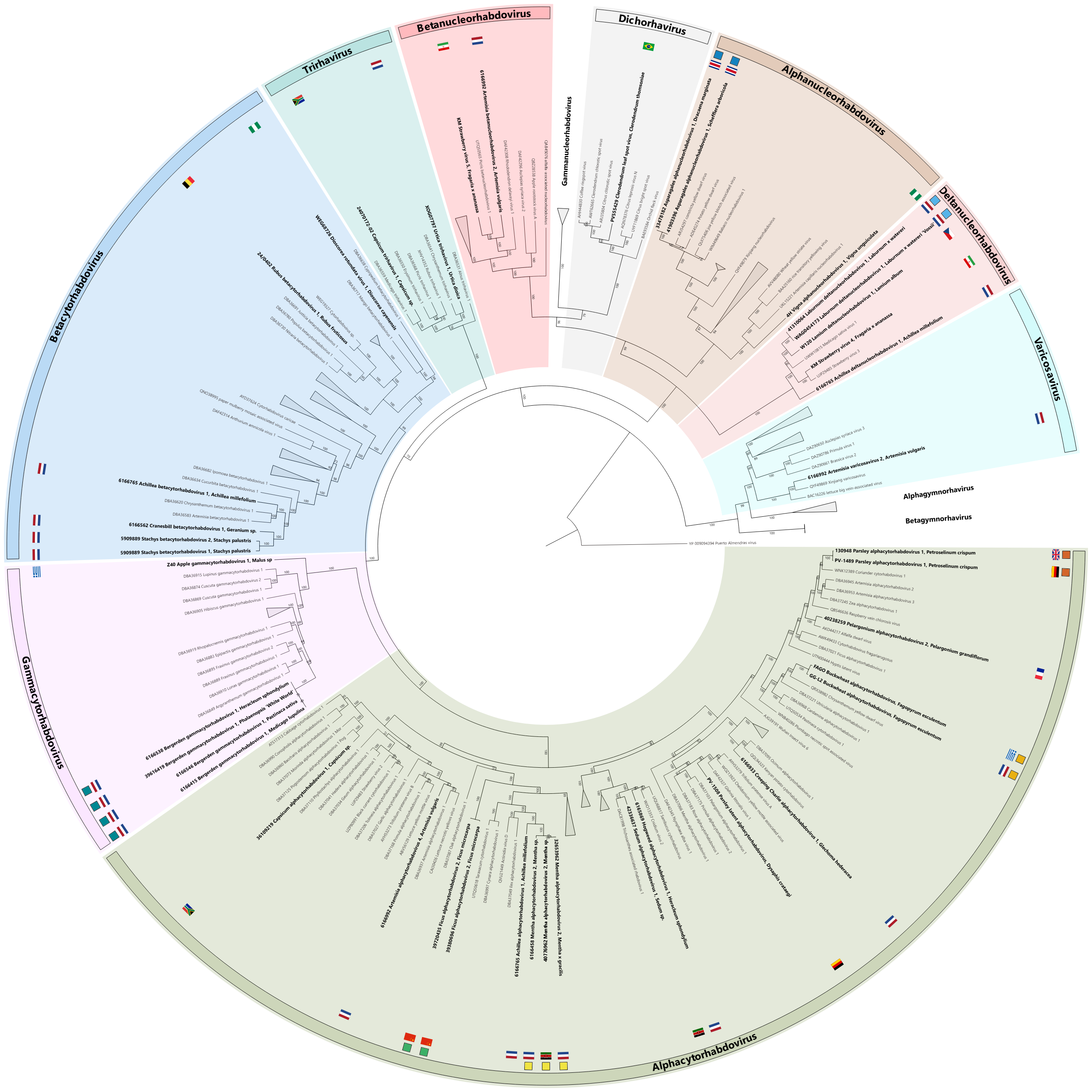

Supplement: Supplementary file 2 — Supplemental Figure S1. Maximum likelihood phylogenetic tree based on the L (RNA-dependent RNA polymerase; RdRp) amino acid sequences. Bold: names of novel viruses. Not bold: NCBI accession numbers representing reference sequences of known rhabdoviruses. Collapsed: clades without novel virus sequences. Colored boxes: identical colors correspond to the same species. Flags mark the country of origin. The tree was constructed using IQ-TREE 2 using the LG+F+I+G4 substitution model and 10,000 bootstrap replicates. Bootstrap support values indicate the percentage of replicate trees in which the associated clade is recovered, reflecting the robustness of the inferred branching. The L protein of Puerto Almendras virus (YP_009094394) was included as an outgroup. (PNG 1.79 MB) [file 705_2026_6609_MOESM2_ESM.png]
