# Supplementary material for: Expanding insights into plant rhabdovirus diversity through the discovery of viruses representing 32 putative novel species
Source: Arch Virol. 2026 Apr 9;171(5):156. doi: 10.1007/s00705-026-06609-1 (PMC13061796; doi:10.1007/s00705-026-06609-1)
Supplement: Supplementary file 3 — Supplemental Figure S2. Bacilliform-shaped particle characteristic of rhabdoviruses observed by transmission electron microscopy in Nicotiana benthamiana infected with Buckwheat alphacytorhabdovirus (sample GG-L2). [file 705_2026_6609_MOESM3_ESM.docx]

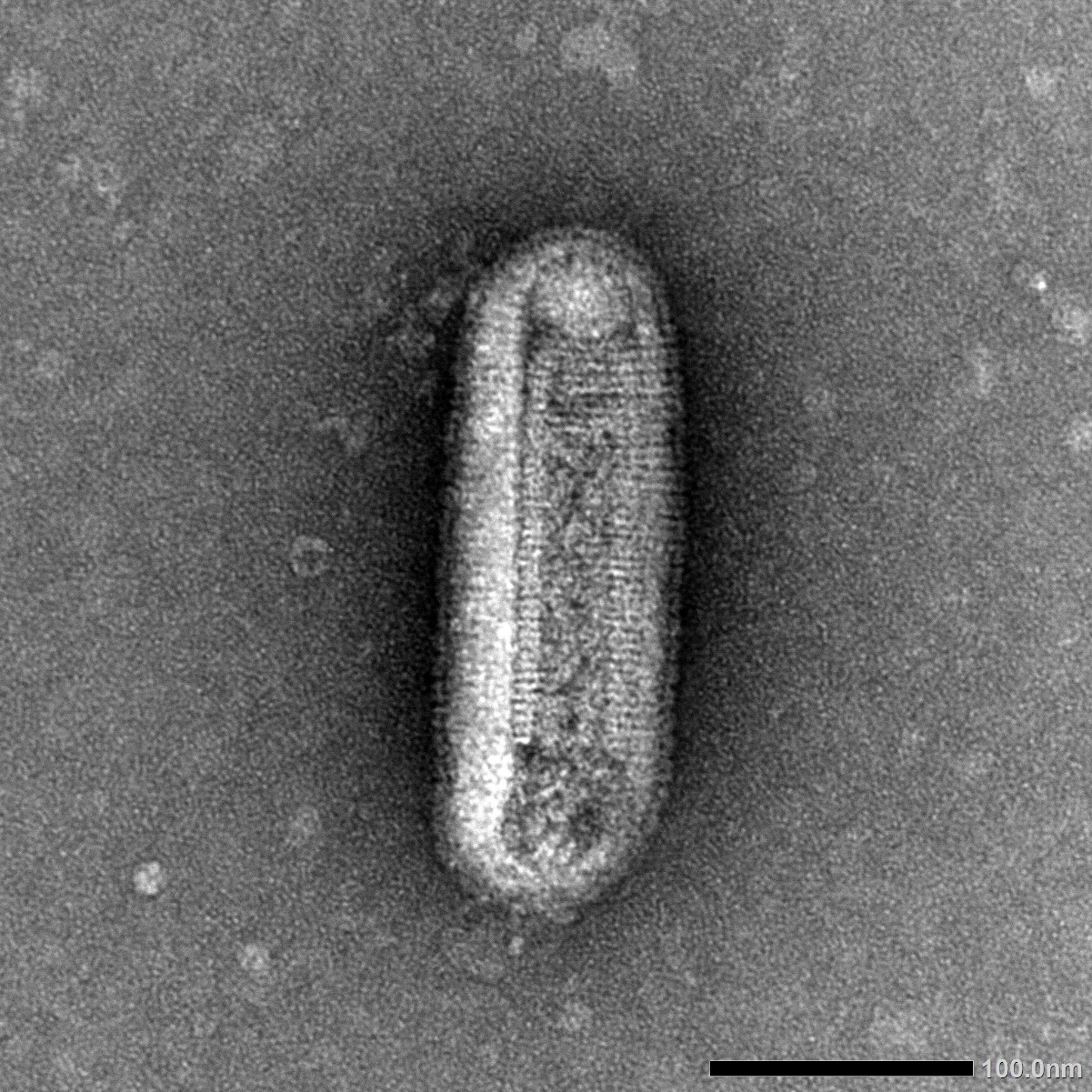


**Supp Fig S2** Bacilliform-shaped particle characteristic of rhabdoviruses observed by transmission electron microscopy in *Nicotiana benthamiana* infected with Buckwheat alphacytorhabdovirus (sample GG-L2).
